# Inflammasome Regulation: Therapeutic Potential for Inflammatory Bowel Disease

**DOI:** 10.3390/molecules26061725

**Published:** 2021-03-19

**Authors:** Qiuyun Xu, Xiaorong Zhou, Warren Strober, Liming Mao

**Affiliations:** 1 Department of Immunology, School of Medicine, Nantong University, 19 Qixiu Road, Nantong 226019, China; qiuyunxu2020@ntu.edu.cn (Q.X.); zhouxiaorong@ntu.edu.cn (X.Z.); 2 Mucosal Immunity Section, Laboratory of Clinical Immunology and Microbiology, National Institute of Allergy and Infectious Diseases, National Institutes of Health, Bethesda, MD 20892, USA; 3 Basic Medical Research Center, School of Medicine, Nantong University, Nantong 226019, China

**Keywords:** inflammasome, inflammatory bowel disease, NLRP3, NLRP6, Crohn’s disease, ulcerative colitis, IL-1β, natural product

## Abstract

Inflammasomes are multiprotein complexes formed to regulate the maturation of pro-inflammatory caspases, in response to intracellular or extracellular stimulants. Accumulating studies showed that the inflammasomes are implicated in the pathogenesis of inflammatory bowel disease (IBD), although their activation is not a decisive factor for the development of IBD. Inflammasomes and related cytokines play an important role in the maintenance of gut immune homeostasis, while its overactivation might induce excess immune responses and consequently cause tissue damage in the gut. Emerging studies provide evidence that some genetic abnormalities might induce enhanced NLRP3 inflammasome activation and cause colitis. In these cases, the colonic inflammation can be ameliorated by blocking NLRP3 activation or its downstream cytokine IL-1β. A number of natural products were shown to play a role in preventing colon inflammation in various experimental colitis models. On the other hand, lack of inflammasome function also causes intestinal abnormalities. Thus, an appropriate regulation of inflammasomes might be a promising therapeutic strategy for IBD intervention. This review aims at summarizing the main findings in these studies and provide an outline for further studies that might contribute to our understanding of the role of inflammasomes in the pathogenesis and therapeutic treatment of IBD.

## 1. Introduction

### 1.1. Inflammatory Bowel Disease

Inflammatory bowel disease (IBD) is a chronic, relapsing disorder affecting the gastrointestinal (GI) tract [1,2]. IBD includes two main subtypes, Crohn’s disease (CD) and ulcerative colitis (UC). CD might affect any part of the GI tract but is most commonly found in the terminal ileum, cecum, peri-anal area, and the colon; it is marked by a dense inflammatory cell infiltration affecting all layers of the bowel wall. In contrast, inflammation in UC only affects the mucosal layer of the colon, causing infiltration of inflammatory cells [1]. IBD occurs in people of all ages with increasing incidence and prevalence worldwide [3]. Chronic colonic inflammation also increases the risk of developing colorectal cancer and, overall, causes serious social and economic burden [4,5,6].

Despite recent advances in our understanding of IBD, the exact pathogenesis of this disease remains elusive. A growing body of evidence supports the hypothesis that the onset of IBD is due to the interplay between host genetic, and environmental factors that cause an imbalanced mucosal homeostasis marked by an inappropriate immune response in the gut [7,8,9]. The innate immune system provides the first line of the host defense against pathogenic microbes and also plays an important role in regulating the immune homeostasis of the intestine [10,11,12]. As a major arm of innate immunity, the inflammasomes are considered to be prominent factors in modulating the development of inflammatory responses in the gut [13,14]. Mounting recent evidence shows that inflammasomes might have the potential to be a therapeutic target for treatment of IBD.

### 1.2. The Inflammasomes

Inflammasome is an intracellular multiprotein complex formed in response to the sensing of pathogen-associated molecular patterns (PAMPs) derived from invading pathogens, or danger-associated molecular patterns (DAMPs) derived from stress or injured cells. The concept of inflammasome was first proposed by Tschopp et al. in 2002 when they studied the function of NALP1 in regulating proinflammatory caspase activation and IL-1β maturation [15]. Canonical inflammasome assembly is initiated by the activation of an NALP (such as NLRP3 or AIM2) via its pattern recognition receptor, which then aggregates with an apoptosis-associated, speck-like protein containing a caspase recruitment domain (ASC) and caspase-1, to form a platform for caspase-1 activation [16,17,18,19,20]. In contrast, non-canonical inflammasome activation is initiated by cytosolic LPS or Gram-negative bacteria, which directly activates caspase-4 (caspase-11 in mice) and caspase-5. Activated caspase-1 induces the cleavage (i.e., maturation) of cytokine precursors (pro-IL-1β and pro-IL-18) as well as the cleavage of gasdermin D, which forms cell membrane pores that allow the release of the mature IL-1β and IL-18 from the cell. Gasdermin D is also acted on by caspase 4/5 (generated from the non-canonical inflammasome) and this also results in cleaved gasdermin D that mediates pore formation. Such a pore formation can lead to a form of cell death called pyroptosis [21,22,23].

To date, a number of inflammasomes are identified, including NLRP1, NLRP3, NLRC4, AIM2, and Pyrin [19,24,25,26,27]. Among these inflammasomes, the NLRP3 inflammasome is the most studied. Its activation requires two signals—the first signal (signal 1) consists of stimulation of membrane receptors such as TLRs and TNFR that prime NLRP3 and other inflammasome components by inducing their transcriptional expression and appropriate post-translational modification. The second signal (signal 2) consists of a variety of stimuli such as extracellular ATP or the ionophore nigericin that induce assembly of the inflammasome described above. The NLRP3 inflammasome can be activated by both PAMPs and DAMPs. Thus, it is not only implicated in host protective immune responses against invading pathogens, but also in the recognition of various endogenous danger signals that regulate the pathogenesis of a variety of human diseases, such as cancer and autoimmune diseases, including IBD [28,29,30].

### 1.3. The Inflammasomes Are Implicated in the Pathogenesis of IBD

Although the inflammasome processes IL-1β—a pro-inflammatory cytokine that is involved in the pathogenesis of many human diseases—it is not a decisive factor for ordinary IBD, since ordinary IBD patients do not respond to treatment by IL-1β blockade. In addition, patients with cryopyrin-associated periodic syndrome (CAPS) carrying a NLRP3 variant with a lowered activation threshold do not develop colonic symptoms indicative of IBD [31,32,33]. Nevertheless, previous studies showed that the production of IL-1β is increased in the serum of IBD patients and is associated with increased disease activity [34,35], indicating some level of involvement of inflammasomes and IL-1β in IBD. However, their precise contributions are controversial, at least for the NLRP3 inflammasome [36,37,38,39]. This is related to various factors, such as the genetic background of the mice or humans studied, the composition of the gut microbial community and the selection of the colitis model [28,40]. It is possible that inflammasome activation might contribute to the development of gut homeostasis in two ways. On one hand, activation that is regulated might play a protective role in the maintenance of gut immune balance; on the other hand, unregulated and excess activation might result in tissue damage and cause colitis [13,41]. In this review, we will discuss available studies focusing on the role of the inflammasomes in the maintenance of gut homeostasis, the development of IBD, as well as the application of therapeutic inhibition of inflammasomes or their downstream cytokines in treatment of IBD in both patients and animal models.

## 2. NLRP3 Inflammasome Activation Contributes to Gut Homeostasis

Many recent studies showed that the NLRP3 inflammasome is involved in regulating colonic immune homeostasis. A good example of this is the findings reported by Huber et al. [42] showing that the NLRP3 and NLRP6 inflammasome-mediated production of IL-18 in the colon promotes the downregulation of IL-22 binding protein (IL-22BP), a soluble receptor expressed by immune cells that specifically binds to IL-22 and blocks the role of IL-22 in regulating colon epithelial cell repair. The epithelial repair function of IL-18 was also supported by studies of Zaki et al. [38], showing that mice with deficiency of NLRP3 inflammasome components exhibit increased Dextran Sodium Sulfate (DSS)-colitis and decreased epithelial integrity, as compared to the wild-type mice. Epithelial cells from these mice produced reduced levels of IL-18, and exogenous IL-18 reversed the increased colitis.

An NLRP3 inflammasome protective role also derives from studies using mice, carrying various genetic abnormalities affecting inflammasome regulators. For instance, Song et al. [43] showed that mice with absence of an E3 ubiquitin ligase, TRIM31, displayed enhanced NLRP3 inflammasome activation and reduced DSS-colitis. Similarly, Cui et al. [44] found that mice with macrophage-specific deficiency of CD1d1 also exhibited enhanced NLRP3 inflammasome activation and reduced DSS-colitis. The mechanism here might be that this deficiency enhances peroxiredoxin 1-associated ATK-STAT1 phosphorylation and subsequent NF-kB activation, and the latter causes increased transcription of NLRP3, IL-1β, and IL-18 in macrophages. Finally, abnormalities of protein tyrosine phosphatase non-receptor 22 (PTPN22) activity were shown to be inversely associated with colitis. Thus, Spalinger et al. [45] reported that PTPN22 deficiency causes pronounced colitis that is associated with inhibited inflammasome activation and IL-1β maturation due to increased NLRP3 phosphorylation at Tyr861. In contrast, an autoimmunity-associated PTPN22 variant (V619W) that leads to increased PTPN22 function causes increased NLRP3 inflammasome activation and excess IL-1β production that protects mice from experimental colitis. Thus, in these cases, upregulating the activity of the NLRP3 inflammasome might be a way to prevent the colonic inflammation.

## 3. Inhibition of the NLRP3 Inflammasome Ameliorates Colitis

**Figure 1:**
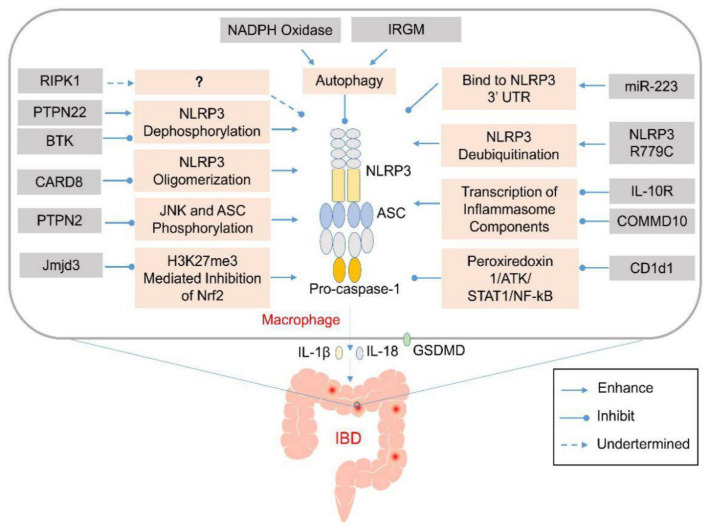
Genetic abnormalities affect development of inflammatory bowel disease (IBD) by elevating the activation of inflammasomes via various cellular processes. Various genetic factors might prevent the activation of the NLRP3 inflammasome via various mechanisms that affect NLRP3 oligomerization or modulate autophagy. Their absence might induce aberrant activation of the NLRP3 inflammasome and excess production of IL-1β and IL-18 in the infiltrating macrophages or dendritic cells in the lamina propria of the colon. The released pro-inflammatory cytokines and pyroptosis-mediated release of cellular contents might recruit other inflammatory mediators to promote the expansion of the colonic inflammation.

The view that the NLRP3 inflammasome has a protective role in colitis, supported by the studies described above, is challenged by other studies showing that loss of this inflammasome function is associated with decreased colitis by one of several mechanisms, including decreased production of potentially damaging pro-inflammatory cytokines such as IL-18 or loss of suppressor cell development [46,47]. In addition, this view is inconsistent with a growing body of studies of various genetic abnormalities showing that excess inflammasome activation causes intensified colitis (summarized in Figure 1 and Table 1). In addition, the protective effect ascribed to NLRP3 inflammasome activation is not supported by many studies with animal models of colitis, which indicate that certain chemicals from natural products, microbes, probiotics, and stem-cell-based therapies prevent colonic inflammation by regulating the function of inflammasomes. Here, we summarize these studies and discuss their possible clinical applications to IBD.

### 3.1. Genetic Factors

Immunity-related GTPase family M (IRGM). IRGM is a component of the autophagy initiation complex that was shown to be implicated in Crohn’s disease risk [48]. A study conducted by Mehto et al. [49] showed that IRGM interacts with NLRP3 and mediates autophagic degradation of NLRP3, thus inhibiting inflammasome assembly. Thus, IRGM-deficient mice showed increased NLRP3 inflammasome activation in the colon and increased DSS-induced colitis. Moreover, administration of the NLRP3 inhibitor, MCC950, ameliorated the intensified colitis in IRGM-deficient mice. Whether NLRP3 inhibition benefits the IBD patients with IRGM deficiency is worthy of investigation.

Receptor interacting protein kinase 1 (RIPK1). RIPK1 is a multitasking kinase that regulates cell death and inflammation [50]. Li et al. [51] investigated the role of RIPK1 in colitis and found that RIPK1 deficiency causes reduced NF-kB activation, defective differentiation of T and B cells, and increased NLRP3 inflammasome activation.

Patients with RIPK1 deficiency manifest combined immunodeficiency and IBD. Whether the colonic inflammation in these patients is caused by increased NLRP3 inflammasome activation and IL-1β generation is not yet determined.

Deficiency of Bruton’s tyrosine kinase (BTK), a cause of X-linked agammaglobulinemia (XLA), is sometimes associated with colonic inflammation [52]. Mao et al. [53] studied the role of BTK in XLA-associated colitis and showed that BTK deficiency augments NLRP3 inflammasome activation by regulating PP2A-mediated NLRP3 pyrin domain dephosphorylation. BTK-deficient mice show increased DSS- or 2,4,6-trinitrobenzene sulfonic acid (TNBS)-induced colitis, which can be ameliorated by administration of IL-1β monoclonal antibody. These findings suggested a possible role of IL-1β blockade in treating colon inflammation in XLA patients with colitis.

Deficiency of protein tyrosine phosphatase non-receptor type 2 (PTPN2) causes early death in mice due to systemic inflammation and severe colitis. Spalinger et al. [54] found that myeloid cell-specific deficiency of PTPN2 induces intensified DSS-induced colitis in mice, while it protects mice from colitis-associated cancer. Regarding the mechanisms involved, PTPN2 deficiency elevated JNK activation, ASC phosphorylation, and enhanced activation of NLRP3 and AIM2 inflammasome, and consequently, increased production of IL-1β. Moreover, inhibition of IL-1β rescued mice from increased colitis in PTPN2-deficient mice. Together, PTPN2 plays an inhibitory role in the development of colitis by regulating inflammasome activation and IL-1β production.

The Jumonji domain-containing 3 (Jmjd3) is an epigenetic regulator that is involved in inflammatory responses. Huang et al. [55] investigated the role of Jmjd3 in development of colitis. They found that Jmjd3 inhibitor (GSK J4) or knock-down significantly downregulated NLRP3 inflammasome activation, by promoting the recruitment of H3K27me3 to the Nrf2 promotor, and thus preventing activation of Nrf2. Moreover, oral administration of the Jmjd3 inhibitor ameliorated the severity of DSS-induced colitis in mice and inhibited the activation of NLRP3 inflammasome in the colon. Thus, Jmjd3 might be a potential target for treatment of IBD, probably by regulating the activation of Nrf2 and the NLRP3 inflammasome.

miR-223 is microRNA that plays a role in regulation of infection, inflammation, and cancer. In studies by Neudecker et al. [56], the role of microRNA miR-223 in IBD was investigated. The data showed that the expression of miR-223 was increased in biopsies from active IBD patients and that miR-223-deficient mice exhibit exacerbated experimental DSS-colitis characterized by increased NLRP3 inflammasome activation, and elevated IL1β production in the colon. In studies of the mechanism involved, they showed that miR-223 prevented activation of NLRP3 inflammasome by binding to the NLRP3 3’ untranslated region. Then, in in vivo experiments, they showed that the intensified colitis could be ameliorated by depletion of CCR2+ inflammatory monocytes, and that overexpression of miR-223 caused blockade of IL-1β or NLRP3. Thus, these findings demonstrated that miR-223 prevents intestinal inflammation by constraining the NLRP3 inflammasome.

As mentioned in the Introduction, NLRP3 variants found in CAPS do not predispose individuals to IBD. Nevertheless, it was shown in a number of studies that NLRP3 polymorphisms were associated with CD or UC [57,58,59,60]. A study by Zhou et al. [61] identified a dominant gain-of-function missense mutation in NLRP3 (R779C), in patients with very-early-onset inflammatory bowel disease (VEOIBD), a chronic inflammatory condition affecting the gastrointestinal tract during infancy or early childhood. They showed that R779C mutation increased NLRP3 inflammasome activation and pyroptosis, through enhanced deubiquitination of NLRP3, via binding with deubiquitinases BRCC3 and JOSD2 in macrophages. Using the DSS-colitis model, they found that mice bearing NLRP3-R779C mutation in hematopoietic cells showed intensified colitis, which was ameliorated by downregulation of BRCC3 or JOSD2. Thus, the R779C variant promotes the risk of developing VEOIBD by elevating the activation of the NLRP3 inflammasome. This suggests that downregulating the expression of BRCC3 or JOSD2 might be a potential therapeutic strategy for this disease.

COMMD family proteins play a role in inflammation by regulating protein trafficking events. Mouhadeb et al. [62] showed that COMMD10 was a key regulator of Ly6C^hi^ monocyte-mediated inflammation and its deficiency in Ly6C^hi^ monocytes caused enhanced production of IL-1β and aggravated colon inflammation in the DSS-induced colitis model. The expression of COMMD10 was decreased in Ly6C^hi^ monocytes from DSS-treated mice and human CD14+ monocytes from IBD patients. Thus, data from this study suggest that COMMD10 is a negative regulator of the inflammasome in monocytes, in patients with IBD.

The studies mentioned above demonstrated that the NLRP3 inflammasome can work as a target for treating colon inflammation in animal models with particular genetic abnormalities. However, investigation in IBD patients might provide more decisive evidence regarding the contributions of inflammasome in IBD. Mao et al. [63] identified a loss-of-function mutation of CARD8 that exhibits defective inhibition of NLRP3 inflammasome and causes IL-1β-mediated CD. The proband patient showed an increased serum level of IL-1β and responded to treatment via blocking IL-1β. In mechanistic studies, Mao et al. showed that the mutation was located in the CARD8 T60 isoform and this mutated isoform blocked NLRP3 oligomerization upon binding to the latter. This study provided definitive evidence that NLRP3 inflammasome and excessive IL-1β production due to a genetic abnormality can be a cause of CD.

IL-10R deficiency or loss-of-function mutations cause spontaneous colitis in mice and infant-onset IBD in humans. Shouval et al. [64] showed that transfer of Il1r1^−/−^ CD4^+^ T cells into Rag1^−/−^/Il10rb^−/−^ mice reduced the severity of colitis in these mice, demonstrating a key role of IL-1β produced by innate immune cells, in the development of colitis in IL-10R-deficient mice. IL10 reduces canonical activation of the NLRP3 inflammasome and production of IL1β at both the transcriptional and post-translational level in wild-type macrophages. Thus, IL-10R deficiency caused an increase in activation of the NLRP3 inflammasome and IL-1β production. LPS alone triggered IL1β secretion in human IL10R-deficient macrophages via non-canonical, caspase 8-dependent activation of the inflammasome. More importantly, two patients with refractory infant-onset IBD responded to the treatment of anakinra, an IL-1-receptor antagonist. Thus, these studies suggested that IBD due to IL-10R deficiency might be treated by blocking IL-1 signaling.

NADPH oxidase deficiency is a cause of colitis found in patients with chronic granulomatous disease (CGD). De Luca et al. [65] showed that inflammatory cells in CGD mice and CGD patients exhibit defective autophagy, increased inflammasome activation and IL-1β release. Compared with wildtype mice, the CGD mice developed increased TNBS-induced colitis, which can be ameliorated by blocking IL-1 signaling with anakinra, an IL-1 receptor antagonist. Anakinra restored autophagy and decreased the activation of the NLRP3 inflammasome. Anakinra treatment also reduced the severe colitis in CGD patients. Thus, CGD-associated colitis can be blocked by regulating IL-1β signaling.

Together, the studies described above demonstrate that excessive activation of the NLRP3 inflammasome and IL-1-mediated signaling play a detrimental role in colitis. Targeting the inflammasome and related cytokines or in combination with other medicines that block other inflammatory pathways generated by particular genetic abnormalities might be a promising strategy for the treatment of IBD.

### 3.2. Natural Products

**Figure 2:**
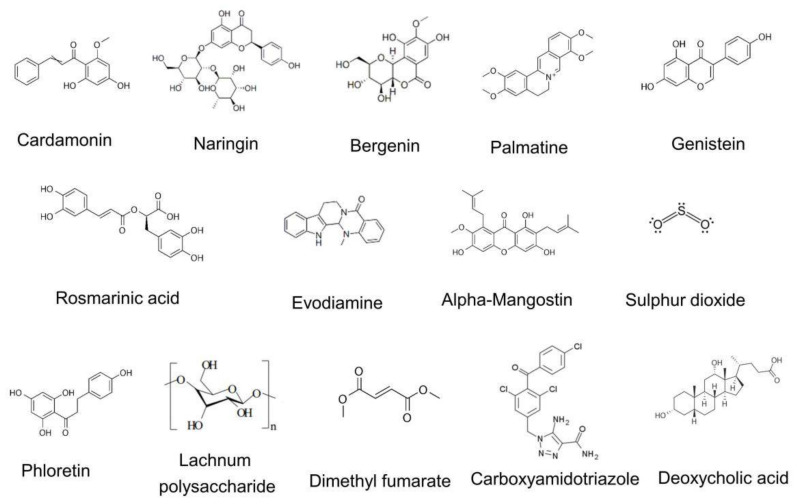
The molecular structure of the chemical compounds that inhibits the activation of the NLRP3 inflammasome. The molecular structures of compounds are diverse; thus these compounds might employ multiple mechanisms in preventing the activation of the NLRP3 inflammasome.

IBD is characterized by chronic and refractory inflammation, thus appropriate inhibition of inflammatory response in the colon might be the key to the development of new anti-inflammatory drugs with greater efficiency and lower side effects. Mounting evidence shows that some natural products might affect the severity of colonic inflammation in various colitis models by targeting the NLRP3 inflammasome (summarized in Table 2). The diversity in molecular structures of these chemical compounds indicates that multiple mechanisms are employed by these compounds in regulating the activation of the NLRP3 inflammasome (Figure 2).

Cardamonin, a cardamon-derived chalcone, has a role in regulating infection, cancer, and inflammatory diseases [66]. Wang et al. [67] investigated its impact on development of IBD. They found that cardamonin inhibited NLRP3 inflammasome activation by activating the AhR/Nrf2/NQO1 pathway. In an in vivo study, oral administration of cardamonin ameliorated the severity of DSS-induced colitis and TNBS-induced colitis. Thus, the effect of cardamonin on GI inflammation might be due to its inhibition of the activation of the NLRP3 inflammasome.

Naringin, a flavonoid extracted from citrus fruits, has an antioxidant and anti-inflammatory role [68]. Cao et al. [69] evaluated the role of naringin in the pathogenesis of ulcerative colitis. Using the DSS-induced colitis model, these authors showed that naringin administration relieved DSS-induced disease activity. Mechanistically, naringin activated DSS-induced peroxisome proliferator-activated receptor γ (PPARγ) and suppressed NF-kB activation. The role of naringin can be largely blocked by the PPARγ inhibitor GW9662. Naringin can also prevent the activation of MAPK and NLRP3 inflammasome. Thus, NLRP3 inflammasome is one of the targets of naringin in preventing DSS-induced colitis.

Bergenin is a bioactive metabolite of a number of herbal plants that shows an anti-tumor and anti-inflammatory role [70]. Lopes de Oliveira et al. [71] investigated the role of natural product bergenin in a rat model of colitis induced by TNBS. They found that bergenin administration decreased macroscopic and microscopic damage, reduced neutrophil infiltration, and downregulated COX-2, iNOS, IkB-α, and pSTAT3 protein expression in the inflamed colon. Moreover, bergenin reduced production of IL-1β, IFN-γ, and IL-10 and inhibited NLRP3/ASC inflammasome activation in the colon. Thus, the role of bergenin in amelioration of TNBS-colitis in the rat was, at least in part, dependent on its inhibition on the NLRP3/ASC inflammasome.

Palmatine is a natural occurring isoquinoline alkaloid with various biological activities [72]. Mai et al. [73] assessed its role in the development of colon inflammation. Their results showed that palmatine treatment ameliorated DSS-induced colitis in mice. This was accompanied by a reduced production of MPO, IL-1β, TNF-α, and the infiltration of F4/80+ cells in the colon. Moreover, palmatine suppressed NLRP3 inflammasome activation, but elevated the expression of mitophagy-associated proteins such as LC3, PINK1, and Parkin, in the colon. In in vitro studies, they showed that palmatine inhibited NLRP3 inflammasome activation and promoted expression and recruitment of PINK1 and Parkin to mitochondria in THP-1 differentiated macrophages. Furthermore, cyclosporin A (CysA), a mitophagy inhibitor or PINK1-siRNA, blocked the effect of palmatine in THP-1 cells. The therapeutic effect of palmatine on DSS colitis was also blocked by treatment with CysA. Thus, palmatine inhibited DSS-induced colitis by promoting PINK1/Parkin mediated mitophagy, and consequently the inactivation of the NLRP3 inflammasome in macrophages.

Genistein is a major isoflavone isolated from various plants. It has anti-inflammatory roles [74] and might affect the development of colitis. Chen et al. [75] assessed the role of the NLRP3 inflammasome in the mechanism of the effect of genistein on colitis. Their data showed that genistein treatment attenuated body weight loss, colon shortening, and infiltration of inflammatory cells into the colon in DSS-treated mice. In addition, in PMA-differentiated THP-1 cells and U937 cells, genistein downregulated caspase-1 and IL-1β production, but upregulated intracellular cAMP levels. The latter then interacted with NLRP3 and inhibited inflammasome activation. Genistein lost its role in inhibiting NLRP3 inflammasome activation in TGR5-silenced U937 cells. Therefore, this study suggested that genistein could be used in treating IBD by inhibiting NLRP3 inflammasome activation via TGR5-cAMP signaling in macrophages.

α-mangostin (α-MG) is a herbal medicine isolated from the pericarp of mangosteen fruits that has anti-inflammatory effects [76]. In a study conducted by Yin et al. [77] its role in a rat model of colitis induced by LPS was evaluated. They showed that administration of α-MG ameliorated LPS-induced intestinal villi detachment, congestion, and hemorrhage. In addition, it reduced epithelial cell nuclei deformation and mitochondria swelling. Finally, their data showed that α-MG reduced the impact of LPS on promoting the expression of NLRP3, caspase 1, IL-18, and IL-1β. Thus, α-MG might work as a therapeutic drug for IBD, probably by inhibiting the expression of the NLRP3 inflammasome.

The development of IBD is associated with dysfunction of gut microbiota. It was shown that the abundance of butyrate-producing bacterium *Roseburia intestinalis* (R.I.) was decreased in patients with IBD and might play an anti-colitis role in the DSS-induced colitis model. Wu et al. [78] investigated the role of R.I.-derived flagellin in a colitis model induced by DSS. Their data showed that R.I flagellin treatment ameliorated colitis induced by DSS, inhibited NLRP3 inflammasome activation in the colon, and reduced Gasdermin D-mediated pyroptosis by regulating the expression of miR-223-3p. Thus, R.I flagellin might benefit IBD patients by downregulating the NLRP3 inflammasome activation.

Endogenous sulfur dioxide (SO_2_) is generated by the metabolism of sulfur-containing amino acids, it was previously considered to be a toxic gas, but recent studies showed that it has anti-inflammatory and antioxidant properties. Banerjee et al. [79] studied the role of SO_2_ in the TNBS-induced colitis model in rat and found that SO_2_ administration ameliorated disease severity and inflammasome activation, and this was associated with reduced oxidative stress, ER stress, and autophagy. In contrast, inhibition of SO_2_ production exacerbated colitis. Therefore, the inhibition of inflammasome might be a mechanism of SO_2_-mediated anti-colitis activity.

Phloretin, a dihydrogen chalcone flavonoid isolated from many fruits and vegetables, show antioxidant and anti-inflammatory effects. Zhang et al. [80] investigated the role of phloretin in IBD. Their data showed that the treatment of phloretin protected mice from DSS-induced colitis in mice and reduced the production of pro-inflammatory cytokines in the colon. To explore the mechanisms involved, they showed that phloretin suppressed NF-kB activation, peroxisome proliferator-activated receptor γ (PPARγ), and NLRP3 inflammasome activation. Further studies showed that phloretin reduced oxidative stress and altered the expression of tight junction proteins. This study suggested that regulation of the NLRP3 inflammasome activation might be critical to phloretin’s capacity to reduce colitis.

Another study conducted by Zong et al. [81] assessed the role of Lachnum polysaccharide (LEP) in the amelioration of colitis in the DSS-induced mouse model. They showed that dietary LEP treatment ameliorated DSS-induced colon pathology. As for the mechanisms involved, they showed that LEP altered the expression of tight junction components and mucus layer protection-related proteins. Moreover, LEP inhibited the production of pro-inflammatory cytokines by regulating the PPARγ/NF-kB and IL-6/STAT3 pathways and by reducing the infiltration of inflammatory cells into the colon. LEP also plays a role in inhibiting NLRP3 inflammasome activation, ER stress, and oxidative/nitrosative stress in DSS-induced colitis. Thus, these findings suggested that NLRP3 inflammasome is one of the targets of LEP in inhibiting the development of colitis.

Dimethyl fumarate (DMF) is a derivative of the Krebs cycle intermediate fumarate. It shows an anti-inflammatory role and is licensed to treat multiple sclerosis and psoriasis [82]. It was shown that DMF can induce a strong antioxidant response and protect mice from a murine colitis induced by 2,4-dinitrobenzene sulfuric acid (DNBS) [83]. In the DSS-induced colitis model, DMF induced glutathione (GSH) expression, activated Nrf2, and suppressed the production of pro-inflammatory cytokines. In in vitro studies, DMF suppressed the activation of the NLRP3 inflammasome and caspase-1. The effect of DMF on NLRP3 inflammasome is dependent on Nrf2, a factor that reduced mitochondrial ROS production and mitochondrial DNA release [84]. A study performed by Li et al. [85] confirmed these findings and further showed that DMF administration increased antioxidant enzymes and reduced the expression of the inflammatory mediator COX-2.

Carboxyamidotriazole (CAI) is a calcium channel inhibitor initially developed as a non-cytotoxic chemotherapy agent [86]. Du et al. [87] studied the effect of CAI on colon inflammation, using the TNBS-induced colitis model in rats. Their data showed that oral administration of CAI reduced colon disease severity in TNBS-colitis and this reduction was accompanied by reduced activation of NF-kB, as well as reduced activation of the NLRP3 inflammasome and production of pro-inflammatory cytokines in the inflamed colon. Thus, CAI might exert its anti-colitis role by inhibiting the NLRP3 inflammasome and NF-kB signaling.

Deoxycholic Acid (DCA) derived from a high-fat diet was shown to play a role in promoting colonic inflammation in DSS-colitis [88]. This might be due to the fact that it can be recognized as a DAMP by the NLRP3 inflammasome via its engagement of sphingosine-1-phosphate receptor 2 (S1PR2) (a bile acid receptor) and the consequent induction of cathepsin B release [89]. Treatment of mice with a S1PR2 inhibitor or cathepsin B antagonist reduced the production of mature IL-1β and alleviated colonic inflammation intensified by DCA [89].

Rosmarinic acid (RA) is a metabolite derived from the herb Rosmarinus officinalis L., which is widely used in folk medicine. It was shown to have general antioxidant and anti-inflammatory functions [90], as well as the capacity to prevent intestinal inflammation in various animal models [91,92,93]. Marinho et al. [94] combined RA with chitosan/nutriose-coated niosomes to prevent degradation of RA in the digestive tract, and thus improve its beneficial effects. Using this method, they evaluated the effect of RA on DSS-colitis and found that RA treatment reduced disease activity in this model. They also found that the expression of NLRP3, ASC, caspase-1, and IL-1β in the colon was reduced by RA. These changes in inflammasome components might be due to the increased expression of Nrf2 and HO-1 that accompany the administration of RA. Therefore, the effect of RA in ameliorating colitis might be exerted through its inhibition of the expression of NLRP3 inflammasome components.

Evodiamine (EVO) is a bioactive compound extracted from *Evodiae fructus*. A variety of pharmacological activities of EVO were reported, including its anti-tumor and anti-inflammatory effects. Two recent studies found that EVO ameliorated experimental colitis, at least partially, by regulating the activation of the NLRP3 inflammasome. The effect of EVO on the inflammasome was associated with its role in regulating the activity of NF-kand autophagy [95,96]. However, the impact of EVO on NLRP3 inflammasome might be dependent on environmental factors, since a study by Li et al. [97] showed that EVO might enhance anti-bacterial responses by augmenting the activation of NLRP3 inflammasome. Thus, the role of EVO in colitis and its impact on NLRP3 inflammasome activation needs to be further studied.

As described above, a variety of natural products were shown to play a role in ameliorating experimental colitis, at least in part, by regulating the expression or activation of the NLRP3 inflammasome. However, before the clinical application of these natural compounds could be realized, studies to improve the specificity of the compounds and thus to avoid off-target effects need to be performed. Using an appropriate dose or a combination of various compounds might also improve their effectiveness. On this basis, the natural products might be a promising approach to the control of colonic inflammatory diseases.

### 3.3. Stem Cells

Mesenchymal stem cells (MSCs) have an immunomodulatory function and thus are considered to be new candidates for treatment of many autoimmune diseases, including IBD. Park et al. [98] investigated the role of adipose-derived stem cells (ASCs) in DSS-induced mouse colitis and found that the administration of ASCs ameliorated DSS-induced colitis in mice, by downregulating the infiltration of M1 macrophages into the colon tissue. Using a co-culture method, they showed that ASCs promoted differentiation of THP-1 cells to M2 macrophages. After the co-culture with ASCs, the expression of pro-IL-1β and NLRP3 was increased in THP-1 cells, while the secretion of mature IL-1 and IL-18 was decreased. Thus, these findings suggested a role of ASCs in suppressing colitis by modulating the activation of NLRP3 inflammasome. However, further studies are required to clarify the mechanism whereby ASCs inhibit NLRP3 inflammasome activation.

### 3.4. Probiotics

Chung et al. [99] assessed the role of a widely used probiotic *Enterococcus faecalis* in the development of colitis and colitis-associated CRC. They found that pretreatment with heat-killed *E. faecalis* inhibited the fecal content of commensal microbes as well as *P. mirabilis-* or *E. coli*-induced NLRP3 inflammasome activation, caspase-1 activation, and IL-1β maturation in THP-1-derived macrophages. In addition, they found that *E. faecalis* treatment attenuated phagocytosis, a process required for the full activation of the NLRP3 inflammasome. *E. faecalis* administration ameliorated DSS-induced colitis and CRC in wild-type mice, while this effect was not seen in NLRP3-deficient mice. Therefore, *E. faecalis* might have a potential therapeutic use in inhibiting colitis and CRC by modulating NLRP3 inflammasome activation.

### 3.5. Hypoxia

Hypoxia can occur under both physiological and pathological conditions and its impact on inflammation varies with these conditions [100,101]. A study conducted by Cosin-Roger [102] examined the role of hypoxia in colitis and showed that environmental hypoxia ameliorated intestinal inflammation. Furthermore, hypoxia treatment increased the turnover of autophagy protein p62, reduced NF-kB activation, NLRP3 expression, and mTOR-NLRP3 interaction. Thus, hypoxia might regulate the development of colitis by downregulating the NLRP3/mTOR pathway and activating autophagy in the colon of Crohn’s disease patients and mice with colitis.

## 4. Regulation of Inflammasomes Other Than NLRP3 in Colitis

### 4.1. The NLRP6 Inflammasome

The NLRP6 inflammasome is an important player in the maintenance of intestinal homeostasis. Elinav et al. [103] studied the role of NLRP6 in colitis, and showed that NLRP6-deficient mice exhibited exacerbation of DSS-induced colitis, due to the induction of colitogenic microbiota. Using co-housing experiments, they found that the colitogenic microbiota were transferable and can cause intensified colitis in wild-type mice. Following this line, the authors in further studies found that some metabolites generated by the microbiota were able to regulate the host-microbiota interface by modulating NLRP6, IL-18, and anti-microbial peptides in the epithelia. The colitogenic microbiota might regulate AMP balance to favor its own colonization via metabolite-mediated inflammasome inhibition [104]. Thus, restoration of the metabolite–inflammasome–AMP axis might be used in disease intervention.

The susceptibility of NLRP6-deficient mice to chemically-induced colitis was confirmed by Chen et al. [105]. These authors showed that loss of NLRP6 was associated with impaired production of IL-18 and increased epithelial injury. In further studies they found that NLRP6 plays a role in inhibiting the spontaneous colitis in IL-10 deficient mice, probably by inducing the enrichment of *Akkermansia muciniphila*, a bacterium that can promote development of colitis [106].

For the regulation of the NLRP6 inflammasome, Mukherjee et al. [107] found that the assembly of this inflammasome was regulated by deubiquitinase Cyld, which mediates deubiquitination of NLRP6; as a consequence, Cyld inhibits NLRP6-ASC assembly and IL-18 production. Cyld deficiency causes elevated levels of mature IL-18 and severe colonic inflammation following *Citrobacter rodentium* infection.

### 4.2. The NLRP1 Inflammasome

A number of studies investigated the role of NLRP1 in pathogenesis of IBD. The first such study was conducted by Williams et al. [108] who showed that Nlrp1b-deficient mice exhibited intensified colitis induced by DSS as compared to wild-type mice. The increased inflammation in Nlrp1b-deficient mice was correlated with reduced production of IL-1β and IL-18 in nonhematopoietic cells in the colon. However, these results were inconsistent with those reported by Tye et al. [109], showing that Nlrp1 deficiency suppressed DSS-induced colitis by promoting the expansion of beneficial, butyrate-producing *Clostridiales*. Moreover, an activating mutation of NLRP1 aggravated DSS-induced colitis, which was associated with an enhanced Th1 response and an increased IL-18/ IFNγ production in the gut. Further studies demonstrated that the colitis in mice carrying activated Nlrp1 was dependent on IL-18. The expression of NLRP1, IL-18, or IFN-γ, negatively correlated with the abundance of *Clostridiales* in biopsies from the patients with ulcerative colitis. Therefore, the contribution of NLRP1 to colonic inflammation might be associated with the composition of gut microbiota, especially the butyrate-producing *Clostridiales* found in the Tye et al. study.

### 4.3. The Pyrin Inflammasome

Mutations in the pyrin gene (Mefv) is associated with hereditary autoinflammatory disease and IBD. In a study by Sharma et al. [110] it was shown that deficiency of Mefv caused increased DSS-colitis with increased epithelial permeability and loss of tight junction proteins. These manifestations were associated with reduced production of IL-18 in the colon, because administration of recombinant IL-18 increased tight junction protein expression, reduced epithelial permeability, and thus reduced the intensified inflammation of Mefv-deficient mice. Thus, compensation of IL-18 might work as a therapy for patients with Mefv mutation or deficiency, but further clinical studies are required to clarify this issue.

### 4.4. The AIM2 Inflammasome

Ratsimandresy et al. [111] investigated the role of the AIM2 inflammasome in IBD. They showed that AIM2 deficiency causes loss of IL-18 and IL-22BP expression in intestinal epithelial cells, which was followed by the loss of STAT3-mediated expression of antimicrobial peptides. In the DSS-induced colitis model, AIM2 deficiency induced excessive production of IL-22, enhanced activation of STAT3 and Akt, and promoted the expression of AMPs Reg3b and Reg3g. Thus, the AIM2 inflammasome prevents intestinal inflammation by regulating the IL-18/IL-22BP/IL22/STAT3 pathway and AMP expression.

### 4.5. The NLRC4 Inflammasome

NLRC4 is an intracellular flagellin receptor. Carvalho et al. [112] assessed the role of NLRC4 in the development of colonic inflammation. Their data showed that NLRC4 deficiency did not alter the gene expression profile induced by the administration of flagellin. However, NLRC4-deficient mice manifested intensified colitis induced by DSS compared with wild-type mice. In further studies, the investigators showed that NLRC4 deficiency caused increased mortality after infection with Salmonella. Thus, NLRC4 inflammasome-mediated production of IL-1β and IL-18 might be the key factors protecting the host from colonic inflammation and infection.

## 5. Conclusions

Accumulating evidence showed that the NLRP3 inflammasome plays a prominent role in the pathogenesis of IBD. In IBD patients carrying particular genetic abnormalities and various experimental models of colitis, excess activation of NLRP3 inflammasome intensifies the colonic inflammation. Treatment targeting this inflammasome or its downstream cytokines proved to be effective in treating certain patients with disease. On the other hand, the inflammasomes contribute to the maintenance of gut immune homeostasis and the lack of inflammasome function might also cause an inflammatory response in the gut. Thus, an appropriate regulation of inflammasome function is required for the intestinal homeostasis. Overall, the activating status of inflammasomes need to be assessed when considering a therapeutic strategy targeting inflammasomes in the treatment of IBD. With the progress of new technologies such as single cell sequencing and immunotherapy, more factors contribute to development of colitis will be identified and then targeted for the treatment of this disease. A deep understanding of the pharmacological properties of the natural compounds might provide new insights for colitis intervention.

## Figures and Tables

**Table 1:** Genetic factors affect IBD by regulating the activation of inflammasomes.

Gene	Function to Inflammasome	Mechanism	Deficiency or Variant	Variant Impact to Disease	Disease or Model	Reference
IRGM	Inhibit NLRP3 inflammasome assambly	Interact with NLRP3 and Promote NLRP3 autophagic degradation	Deficiency	Exacerbate colitis	DSS-colitis in mouse	[49]
RIPK1	Inhibit NLRP3 inflammasome activation upon LPS stimulation	Not clear	Deficiency	Primary immunodeficiency and/or colitis	Human colitis	[51]
BTK	Inhibit NLRP3 inflammasome activation	Inhibit PP2A mediated NLRP3 dephosphorylation	Deficiency	Exacerbate colitis	DSS-colitis, TNBS-colitis in mouse	[53]
PTPN2	Inhibit NLRP3 inflammasome activation	Inhibit JNK and ASC phosphorylation	Deficiency in myeloid cell	Exacerbate colitis	DSS-colitis in mouse	[54]
Jmjd3	Enhance NLRP3 inflammasome activation	Prevent H3K27me3 mediated inhibition of Nrf2	Inhibition or Knock-down	Ameliorate colitis	DSS-colitis	[55]
miR-223	Inhibit NLRP3 inflammasome activation	Bind to NLRP3 3’ untranslated region to inhibit inflammasome assembly	Deficiency	Exacerbate colitis	DSS-colitis in mouse	[56]
NLRP3 mutation	Enhance NLRP3 inflammasome activation	Enhance deubiquitination of NLRP3 via binding with BRCC3 and JOSD2	R779C	Exacerbate colitis	DSS-colitis in mouse	[61]
COMMD10	Inhibit NLRP3 inflammasome activation	Inhibit transcription of inflammasome components	Deficiency in monocytes	Exacerbate colitis	DSS-colitis in mouse	[62]
CARD8	Inhibit NLRP3 inflammasome assembly	Interact with NLRP3 and inhibit NLRP3 oligomerization	V44I	Exacerbate colitis	Crohn’s disease	[63]
IL-10R	Inhibit NLRP3 inflammasome activation	Inhibit expression of NLRP3 and IL-1β	Deficiency	Spontaneous colitis in mouse and infant-onset IBD in human	Mouse spontaneous colitis and human IBD	[64]
NADPH oxidase	Inhibit NLRP3 inflammasome activation	Induce autophagy	Deficiency	Exacerbate colitis	TNBS-colitis in mouse	[65]
CD1d1	Reduce transcription of NLRP3 inflammasome components	Reduce peroxiredoxin 1/ATK/STAT1 mediated NF-kB signaling	Deficiency in macrophage	Ameliorate colitis	DSS-colitis in mouse	[44]
PTPN22	Enhance NLRP3 inflammasome activation	Induce NLRP3 dephosphorylation at Tyr861	Deficiency	Exacerbate colitis	DSS-colitis in mouse	[45]
			V619W	Ameliorate colitis	DSS-colitis in mouse	[45]

Abbreviations: IRGM: Immunity-related GTPase family M; RIPK1: Receptor interacting protein kinase 1; BTK: Bruton’s tyrosine kinase; XLA: X-linked agammaglobulinemia; PTPN2, 22: protein tyrosine phosphatase non-receptor type 2, 22; Jmjd3: Jumonji domain-containing 3; VEOIBD: Very-Early-Onset inflammatory bowel disease; COMMD10: COMM domain containing 10; CARD8: caspase recruitment domain family member 8; CGD: chronic granulomatous disease; PP2A: serine/threonine protein phosphatase 2A; DSS: Dextran Sodium Sulfate; TNBS: 2,4,6-trinitrobenzene sulfonic acid; PPARγ: peroxisome proliferator-activated receptor γ; SO_2_: sulfur dioxide; and CysA: cyclosporin A.

**Table 2:** Preventing colitis in animal models of colitis or patients with IBD by targeting inflammasomes.

Natural Product	Source	Mechanism	Disease or Model	Reference
Cardamonin	Cardamom	Activate AhR/Nrf2/NQO1 pathway	DSS-colitis and TNBS-colitis in mouse	[67]
Naringin	Citrus fruit	Activate PPARγ and suppresse NF-kB activation	DSS-colitis in mouse	[69]
Bergenin	Plant metabolite	Inhibit COX-2, iNOS, IkB-α, and pSTAT3 expression	TNBS-colitis in rat	[71]
Palmatine	Herbal plant	Elevated mitophagy proteins LC3, PINK1 and Parkin	DSS-colitis in mouse	[73]
Genistein	Plant	Elevate intracellular cAMP	DSS-colitis in mouse	[75]
Rosmarinic acid	**Rosmarinus officinalis* L.*	Reduce the expression of inflammasome components	DSS-colitis in mouse	[94]
Evodiamine	Evodiae fructus	Regulate NF-kB and autophagy	DSS-colitis in mouse	[95,96]
α-mangostin	Mangosteen fruit	Promote expression of NLRP3, caspase 1, IL-18, and IL-1β	LPS-induced colitis in rat	[77]
Flagellin	Roseburia intestinalis	Elevate expression of miR-223-3p	DSS-colitis in mouse	[78]
Sulphur dioxide	Metabolism of sulfur-containing amino acids	Reduce oxidative stress, ER stress and autophagy	TNBS-colitis in rat	[79]
Phloretin	Fruits and vegetables	Suppressed NF-kB, PPARγ and oxidative stress	DSS-colitis in mouse	[80]
Lachnum polysaccharide	Lachnum sp.	ER stress and oxidative/nitrosative stress	DSS-colitis in mouse	[81]
Dimethyl fumarate	Derivative of fumarate	Induced GSH, activate Nrf2 and suppress mitochondrial ROS	DNBS-colitis in mouse	[84]
Carboxyamidotriazole	Noncytotoxic chemotherapy agent	Reduce activation of NF-kB	TNBS-colitis in rat	[87]
Deoxycholic Acid	High fat diet	Promote cathepsin B release by engagement of S1PR2	DSS-colitis in mouse	[89]

Abbreviations: DSS: Dextran Sodium Sulfate; TNBS: 2,4,6-trinitrobenzene sulfonic acid; PPARγ: peroxisome proliferator-activated receptor γ; SO_2_: sulfur dioxide; CysA: cyclosporin A; DNBS: 2,4-dinitrobenzene sulfuric acid; GSH: glutathione; and S1PR2: sphingosine-1-phosphate receptor 2.

## Abbreviations

IBD: inflammatory bowel disease; NLRP3, 1, 6: NOD-like receptor family pyrin domain-containing 3, 1, 6; GI: gastrointestinal; CD: Crohn’s disease; UC: ulcerative colitis; PAMPs: pathogen-associated molecular patterns; DAMPs: danger-associated molecular patterns; ASC: apoptosis-associated speck-like protein containing a caspase recruitment domain (CARD); CAPS: cryopyrin-associated periodic syndrome; IRGM: Immunity-related GTPase family M; RIPK1: Receptor interacting protein kinase 1; BTK: Bruton’s tyrosine kinase; XLA: X-linked agammaglobulinemia; PTPN2, 22: protein tyrosine phosphatase non-receptor type 2, 22; Jmjd3: Jumonji domain-containing 3; VEOIBD: Very-Early-Onset inflammatory bowel disease; COMMD10: COMM domain containing 10; CARD8: caspase recruitment domain family member 8; CGD: chronic granulomatous disease; PP2A: serine/threonine protein phosphatase 2A; DSS: Dextran Sodium Sulfate; TNBS: 2,4,6-trinitrobenzene sulfonic acid; PPARγ: peroxisome proliferator-activated receptor γ; SO_2_: sulfur dioxide; CysA: cyclosporin A; DNBS: 2,4-dinitrobenzene sulfuric acid; GSH: glutathione; S1PR2: sphingosine-1-phosphate receptor 2; and MSCs: Mesenchymal stem cells.
